# Stability of Risk Preferences During COVID-19: Evidence From Four Measurements

**DOI:** 10.3389/fpsyg.2021.702028

**Published:** 2022-02-10

**Authors:** Peilu Zhang, Marco A. Palma

**Affiliations:** ^1^Dyson School of Applied Economics and Management, Cornell University, Ithaca, New York, NY, United States; ^2^Department of Agricultural Economics, Texas A&M University, College Station, TX, United States

**Keywords:** gender differences, risk preferences, COVID-19 pandemic, psychological construct of risk-preference, risk-preference measures, JEL Codes: C9, D81, J1

## Abstract

This article studies the stability of risk-preference during the COVID-19 pandemic. The results differ between risk-preference measurements and also men and women. We use March 13, 2020, when President Trump declared a national state of emergency as a time anchor to define the pre-pandemic and on-pandemic periods. The pre-pandemic experiment was conducted on February 21, 2020. There are three on-pandemic rounds conducted 10 days, 15 days, and 20 days after the COVID-19 emergency declaration. We include four different risk-preference measures. Men are more sensitive to the pandemic and become more risk-averse based on the Balloon Analogue Risk Task (BART). Women become more risk-averse in the Social and Experience Seeking domains based on the results from the Domain-Specific Risk-Taking (DOSPERT) and Sensation Seeking Scales (SSS). Both men's and women's risk-preference are stable during COVID-19 based on a Gamble Choice (GC) task. The results match our hypotheses which are based on the discussion about whether the psychological construct of risk-preference is general or domain-specific. The differential outcomes between incentivized behavioral and self-reported propensity measures of risk-preference in our experiment show the caveats for studies using a single measure to test risk-preference changes during COVID-19.

## 1. Introduction

Risk preferences are a key component of individual decision-making and behavior. The question of whether risk preferences are stable over time or under different contexts has received a great deal of attention in previous literature (Anderson and Mellor, 2009; Schildberg-Hörisch, 2018). COVID-19 emerged in December 2019. Since then, it has spread to more than 200 countries. COVID-19 is arguably one of the deadliest pandemics in human history (Zoumpourlis et al., 2020). COVID-19 may change people's preferences or expectations in various aspects of daily life (Barrios and Hochberg, 2020; Binder, 2020; Chan et al., 2020; Guenther et al., 2021). The purpose of this article focuses on individuals' risk-preference changes during COVID-19.

Based on classical economic theories, preferences (including risk preferences) are stable and unaffected by experience over time (Stigler and Becker, 1977). However, evidence from empirical studies shows that people's risk attitudes can be affected by negative shocks (Bogliacino et al., 2021), such as natural disasters (Eckel et al., 2009; Page et al., 2014; Hanaoka et al., 2018; Kahsay and Osberghaus, 2018; Abatayo and Lynham, 2020), financial crisis (Jetter et al., 2020), and violent events (Callen et al., 2014; Jakiela and Ozier, 2019). COVID-19 has affected the global economy substantially. Many people lost their jobs and business during COVID-19 (Kawohl and Nordt, 2020). Thus, COVID-19 may change the background risk for people by changing the living and working environment and bringing more uncertainties to the life (Gollier and Pratt, 1996; Quiggin, 2003; Guiso and Paiella, 2008). The change in background risk may bring changes in the risk-taking behavior of people (Eeckhoudt et al., 1996; Tsetlin and Winkler, 2005; Lee, 2008). More recently and related to this article, studies on risk-preference comparisons between pre-COVID-19 and on-COVID-19 show mixed results. Angrisani et al. (2020), Lohmann et al. (2020), and Drichoutis and Nayga (2021) find no significant change in risk preferences during COVID-19; Gassmann et al. (2020) and Shachat et al. (2021b) suggest less risk aversion or increased risk tolerance during COVID-19; the results from Harrison et al. (2020) and Li et al. (2021), however, exhibit more risk aversion of subjects under COVID-19; Shachat et al. (2021a) find decreased risk tolerance in the loss domain and less risk aversion in the gain domain. All of these studies use only one (type) measurement of risk-preference, and they all use experiments conducted in 2019 as a pre-pandemic baseline. The subjects are all students.

We advance the literature in three major aspects. First, we use four elicitation methods of risk-preference which are widely used in economics and psychology studies: Balloon Analogue Risk Task (BART) developed by Lejuez et al. (2002), the Gamble Choice (GC) developed by Eckel and Grossman (2008), Domain-Specific Risk-Taking (DOSPERT) developed by Blais and Weber (2006), and Sensation Seeking Scale (SSS) developed by Zuckerman et al. (1964). BART and GC are incentivized behavioral measures, and DOSPERT and SSS are self-reported propensity measures. Frey et al. (2017) found a substantial gap between revealed risk-preference by behavioral measures and stated risk-preference by self-reported measures. Frey et al. (2017) acknowledge that the vast majority of past empirical work on risk-preference has typically used single measures of risk-preference, and suggest that using several instruments to measure risk-reference may reduce measurement error for future empirical work.

Second, we believe the timeline of our study can reflect the most immediate and salient changes in risk preferences by the COVID-19 shock. We implemented an online experiment to verify the robustness of Zhang and Palma (2021) on February 21, 2020, that serves as a baseline (pre-pandemic)^1^. The experiment was about risk-taking behavior with insurance, and it consists of the four risk-preference elicitation methods mentioned above. On March 13, 2020, President Trump declared a national state of emergency. We use March 13, as a time anchor and implemented three rounds of parallel experiments conducted online on March 23, 2020, March 28, 2020, and April 2, 2020, respectively. The three dates were selected to be 10, 15, and 20 days after the state of emergency declaration. Note that on February 21, the effects of COVID-19 had not yet spread widely in the United States, and there were only 19 daily confirmed cases based on the Centers for Disease Control and Prevention (CDC) data tracker. Starting March 13, the number of COVID-19 cases in the U.S. increased exponentially. The new daily confirmed cases on our on-pandemic rounds are 11,400, 20,820, 30,157, respectively (refer to Appendix 7 for the trend of daily cases from February 21 to April 2). We argue that the rounds of our experiment reflect the pre and on-pandemic times. Third, we implement an online experiment with more a general population than students as subjects. All four rounds were conducted using Amazon Mturk. The average age of subjects in our experiment is 41, and all subjects are located in the United States. Subjects of the pre and on-pandemic are from the same population. We do not find significant differences in demographic characteristics between pre and on-pandemic rounds, except that there are only marginal significant differences in age and household size (refer to Appendix 8). In regressions in the result section, we control for those demographic variables.

The original experiment in Zhang and Palma (2021) was conducted in November 2016 to investigate risk-taking behavior under different insurance schemes. The main measurement used to assess risk-taking behavior was the BART. We also included three other risk preferences elicitation methods to validate BART as a measurement of risk-taking behavior. The original experiment contains three treatments varying in the insurance types set in BART. For the pre-pandemic round on February 21, 2020, we implemented the entire original experiment with three treatments; we only ran the “Voluntary Insurance” treatment for the other three on-pandemic rounds. The reason is that the focus of this article is risk-preference changes instead of insurance. For the on-pandemic rounds, we added a question at the end of the experiment about whether participants were in self-isolation or not. Around 81% of subjects answered “Yes.” This to some extent confirms that our subjects were experiencing COVID-19 impacts when they were participating in our on-pandemic rounds.

Gender differences in risk preferences is a well documented phenomenon in the economics literature (Eckel and Grossman, 2008). Economists have tried to use the gender gap in risk preferences to explain persistent gender differences in other domains such as willingness to compete and occupation choice (Niederle and Vesterlund, 2007; Marianne, 2011; Shurchkov and Eckel, 2018). Our results also focus on the heterogenous effects of COVID-19 on men and women's risk preferences. We do not obtain consistent results across all four risk-preference elicitation methods. Based on the BART results, men are more sensitive, and become more risk-averse during COVID-19; there is no change in women's risk-preference according to BART. We find a gender gap in risk preferences in the pre-pandemic period based on BART, however, this difference disappears during the pandemic. In both DOSPERT and SSS, women respond more to COVID-19 compared to men and become more risk-averse in the specific Social and Experience Seeking domains. There are no changes in risk-preference for both men and women according to a GC task (Eckel and Grossman, 2008).

The results are in line with our hypotheses about the stability of risk-preference during COVID-19. The debate about whether risk-preference is general or domain-specific has a long history in psychology and economics (Mata et al., 2018). Recent study shows that the psychological construct of risk-preference includes both general and domain-specific components (Frey et al., 2017). We provide our hypotheses based on the discussion of the nature of risk-preference and different psychological traits captured by different measures in our experiment. We elaborate on the hypotheses in Section 3.

Our results provide new insights to the literature about the stability of risk-preference during shocks from two perspectives. First, different risk-preference measures provide differential results and this depends on what psychological traits (general or domain-specific) the measures captured. Second, there is a gender difference in the stability of risk-preference during shocks.

The rest of the article is organized as follows. Section 2 introduces the four risk-preference measures in detail. Section 3 provides the hypotheses. Section 4 present the experimental design and procedures. Section 5 shows the results. Section 6 discusses the correlations of risk-preference measured by the four methods in our experiment, and Section 7 concludes.

## 2. Risk-Preference Measures

The methods for measuring risk preferences date back to the last century (Officer and Halter, 1968; Dillon and Scandizzo, 1978). Well-established instruments both in psychology and economics (Holt and Laury, 2002; Eckel and Grossman, 2008; Dohmen et al., 2011; Charness et al., 2013; Crosetto and Filippin, 2016) have been designed to prevent potential negative outcomes associated with behavior under risk and uncertainty. In this article, we use four risk-preference elicitation methods: BART, GC, DOSPERT, and SSS.

**Balloon Analogue Risk Task** Participants are presented with a balloon on separate computer screens and they are asked to pump the balloon from 1 to 128 times. The balloon may explode at some point. Participants receive a monetary reward for each successful pump. However, if the balloon explodes, they receive nothing. This means a higher number of pumps carries higher potential earnings but also a higher probability of an explosion. Therefore, a higher number of pumps indicates more risk-taking behavior, and hence BART is able to assess risk-taking behavior by using the average number of pumps. Please refer to the Appendix for the explosion probability algorithm for each balloon. BART allows for the calculation of the risk coefficient for each pump choice under the assumption of constant relative risk aversion (CRRA) (Zhang and Palma, 2021). According to the risk coefficient, risk-averse, risk-neutral, risk-loving individuals choose less than 64, 64, and more than 64 pumps, respectively. In this article, we use the automatic version of BART (Pleskac et al., 2008) in which participants input the number of pumps into a box and the balloon is pumped automatically^2^. Subjects see the process of pumping and the outcome of the balloon before they proceed to the next balloon.

The advantage of BART is that participants do not need extra knowledge to understand the game. The disadvantage of BART is that it needs to be computerized, and there is ambiguity about the probability of explosion (Charness et al., 2013; Crosetto and Filippin, 2013). Thus, we inform subjects that the maximum number of pumps for each balloon is 128 in order to reduce potential ambiguity.

**Gamble Choice** is designed as a simple set of lottery choices that produce enough variance to allow for the estimation of utility parameters and risk preferences. Participants are presented with six gambles and they are asked to choose the one that they would like to play. In Table 1, each of the gambles involves a 50% chance of receiving a high payoff and a 50% chance for a low payoff. The first gamble in our experiment has a certain payoff of 10 cents^3^. For gambles 1-5, both the expected payoffs and SD (risk) increase linearly. Note that gamble 6 has the same expected payoff as gamble 5 but with a higher variance. Under the CRRA assumption, each gamble implies an interval for the risk coefficient with the utility function (*u*(*x*) = *x*^1−*r*^). Risk-averse subjects with *r*> 0 choose gambles 1–4 where the variance is lower. Risk-neutral subjects with *r* = 0 choose gamble 5, which has the highest expected return. Risk-seeking subjects with *r* < 0 choose gamble 6, which has higher expected payoff and a higher variance.

**Table 1:** Gamble choice (GC) task.

**Gamble choice**	**The event**	**Probability(%)**	**Payoff (cents)**
1.	A	50	10
	B	50	10
2.	A	50	18
	B	50	6
3.	A	50	26
	B	50	2
4.	A	50	34
	B	50	–2
5.	A	50	42
	B	50	–6
6.	A	50	44
	B	50	–8

**Domain-Specific Risk-Taking** was developed to take into account that risk attitudes may vary across different domains. For example, people differ in the way they resolve finance-related or health-related decisions in which risk attitudes play a key role. DOSPERT assesses risk-taking behavior in five different content domains: financial, health, recreational, ethical, and social decisions. Financial decisions contain two subcategories: investing and gambling. DOSPERT contains 30 questions in total. Participants are asked to rate the likelihood that they would engage in the specific risky activities for each question using a 7-point rating scale from 1 (extremely unlikely) to 7 (extremely likely). The self-reported risk-taking measures in DOPSERT have been documented to be significantly correlated with risk-taking behavior in the real world in a variety of domains (Farnham et al., 2018; Shou and Olney, 2020). The full questionnaire is shown in the Appendix.

**Sensation Seeking Scale** consists of 40 forced-choice items designed to measure sensation seeking traits as a psychological instrument. A sample item includes “A. I often wish I could be a mountain climber. B. I can't understand people who risk their necks climbing mountains.” Participants must choose one of the two options for each item. The SSS yields one total score and four primary sub-scales with 10 items for each sub-scale: (1) Thrill and Adventure Seeking (TAS, desire to engage in sports or activities involving speed and danger; e.g., mountain climbing), (2) Experience Seeking (ES, desire to experience through the mind and senses, travel, and a non-conforming lifestyle; e.g., dressing in strange ways), (3) Disinhibition (DIS, desire for social and sexual disinhibition; e.g., “uninhibited” parties), and (4) Boredom Susceptibility (BS, aversion to repetition, routine, and dull people; e.g., preference for unpredictable friends). The SSS has been shown to be reliable across cultures, ages, and genders (Zuckerman et al., 1978). The concept of sensation seeking is presumed to account for differences in people's willingness to participate in risky activities across a wide range of behaviors (Zuckerman, 1994). Higher scores in SSS indicate higher risk-taking. The self-reported risk-taking measures in SSS have been found to be associated with risky behavior in different settings (Zaleski, 1984; Wong and Carducci, 1991; Zuckerman, 2007). The description of the scales and item loadings are listed in the Appendix.

## 3. Hypotheses

The four elicitation methods used in our experiment belong to two different measurement traditions of risk preferences in psychology and economics (Charness et al., 2013; Mata et al., 2018). BART and GC are incentivized behavioral measures eliciting the revealed risk preferences. DOSPERT and GC are self-reported propensity measures accessing the stated risk preferences. The question about whether risk-preference should be conceptualized as a general psychological construct, or as domain-specific construct, or as a combination of both has received a great deal of attention in psychology and economics (Zhong et al., 2009; Benjamin et al., 2012; Highhouse et al., 2017). If the nature of risk-preference is a general construct, then risk-preference should be a stable psychological trait across time and domains; if risk-preference is domain-specific, then it includes various traits in different domains such as finance, health, and experience. Frey et al. (2017) used 39 risk-preference measures to study the psychometric structure of risk-preference, and they suggest that the construct of risk-preference contains both general and domain-specific components, with a general factor of risk-preference explaining half of the variance and a series domain-specific factors explaining the other half.

Frey et al. (2017) also suggest a substantial gap between behavioral and propensity risk-preference measurement traditions. Both Frey et al. (2017) and Mata et al. (2018) argue a primacy of self-reported propensity measures over behavioral measures by studying the *temporal stability, convergent validity*, and *predictive validity* of the measures.

COVID-19 affects various aspects of life and work with different levels of degree. For example, people are required to self-isolate and keep social distance during COVID-19, and many companies lay off workers and freeze hiring. People have lower expectations about careers due to COVID-19. Thus, COVID-19 may have stronger effects of those experiences and social-related aspects of life. Different risk-preference measures may capture different psychological traits Mata et al. (2018). In our experiment, the behavioral measures (BART & GC) are more likely to capture a unitary psychological trait that is stable across domains; DOPSERT and SSS contains sub-domains, and they are more likely to capture various domain-specific traits. Thus, we have the following hypotheses.

**Hypothesis 1**. Risk-preference tends to be stable during COVID-19 when it is measured by BART and GC.
**Hypothesis 2**. The risk-preference measured in specific domains by DOSPERT and SSS which are likely to be affected by COVID-19 changes during COVID-19.

## 4. Experimental Design

The experiment was conducted on Amazon's Mechanical Turk (MTurk), an online labor market platform where businesses and individuals can post tasks and workers perform the tasks in exchange for a payment. We published a Human Intelligence Task (HIT) on MTurk. The HIT provided instructions about the type, length, payment, and IRB information for the experiment. The workers on MTurk decided whether they want to participate or not after reading the instruction^4^. The experiment was computerized in Inquisit (Inquisit, 2016). Interested workers were instructed to click on a link included in the HIT that took them to the experiment implemented by the Inquisit web lab. The first page of the experiment was the electronic consent form. Participation was voluntary and workers could decide to quit at any time. For those who completed the experiment, a unique random ID was generated on the last page of the experiment. Workers were required to submit their code through MTurk. We later used these ID- codes to make the payments by linking the codes recoded on our experiment to the workers' MTurk account. Workers' earnings included a fixed participation fee and a bonus from the incentivized BART and GC tasks during the experiment. The average payment was $2.

The experiment consists of four rounds. The first round was conducted on February 21, 2020, which we refer to as the pre-pandemic round. For the pre-pandemic round, we use the entire between-subject treatments in Zhang and Palma (2021). The original design in Zhang and Palma (2021) was between-subjects with three treatment groups varying in insurance schemes set in the BART: Voluntary Insurance, Compulsory Insurance, and Mixed Insurance. In each treatment group, participants were asked to work on the tasks in the following order: BART, DOSPERT, SSS, and GC. A demographic survey was included at the end of the experiment. Each subject was only allowed to participate in one treatment, and hence there is no income or order effect concerns.

Having risk preference data for the pre-pandemic period serves as motivation for this article. Since the focus of this article is about risk-preferences, we only implemented the “Voluntary Insurance” treatment for the on-pandemic rounds. When we compare the pre-pandemic and on-pandemic outcomes, only data of the “Voluntary Insurance” treatment in the pre-pandemic round is used. The three on-pandemic rounds were conducted on March 23, 2020; March 28, 2020; and April 2, 2020.

In BART, subjects played 30 sequential balloons. Subjects received ¢1 for each successful pump for each balloon. For the **first** and **last** balloon, there was an insurance option with a premium of ¢40 and coverage of ¢64. Subjects can voluntarily choose to buy the insurance or not^5^. For the middle **28** balloons, subjects play BART without an insurance option. At the end of the experiment, three balloons are randomly selected to determine the earnings for BART. In this article, we only focus on the middle 28 balloons where risk is measured without insurance options. Note that all four rounds had identical procedures, thus allowing us to make comparisons across rounds.

In total there were 331 subjects. We removed data from 9 subjects who chose 128 in some of the balloons. Choosing 128 pumps guarantees an explosion. Hence, we treat them as not understanding the BART task or not paying attention to the instructions. Thus, the final sample consists of 322 subjects. Table 2 summarizes the experiment and the number of observations for each round. The instructions are available in the Appendix.

**Table 2:** Summary of each round.

	**Treatment**	**BART**	**DOSPERT**	**SSS**	**GC**	**No. Subjects**
Feb 21rd	All three treatments	Middle 28 balloons	30 items	40 itms	6 lotteries	84
	(only data from Voluntary insurance is used)
Mar 23rd	Voluntary insurance	Middle 28 balloons	30 items	40 itms	6 lotteries	82
Mar 28th	Voluntary insurance	Middle 28 balloons	30 items	40 itms	6 lotteries	75
Apr 2nd	Voluntary insurance	Middle 28 balloons	30 items	40 itms	6 lotteries	81

## 5. Results

In this section, we show the results of the four elicitation methods separately starting with the incentivized tasks.

**Balloon Analogue Risk Task**. In BART, subjects play with insurance options in the first and last balloon, and they play the normal balloon without insurance for the middle 28 balloons. Thus, we focus on the average number of pumps of the middle 28 balloons to analyze the risk-taking behavior measured by BART. We find participants become more risk-averse during COVID-19. The average number of pumps in the pre-pandemic round is 60.06, and it is 52.41 in the on-pandemic rounds (Mann-Whitney U-Test, *p* = 0.002).

**Figure 1:**
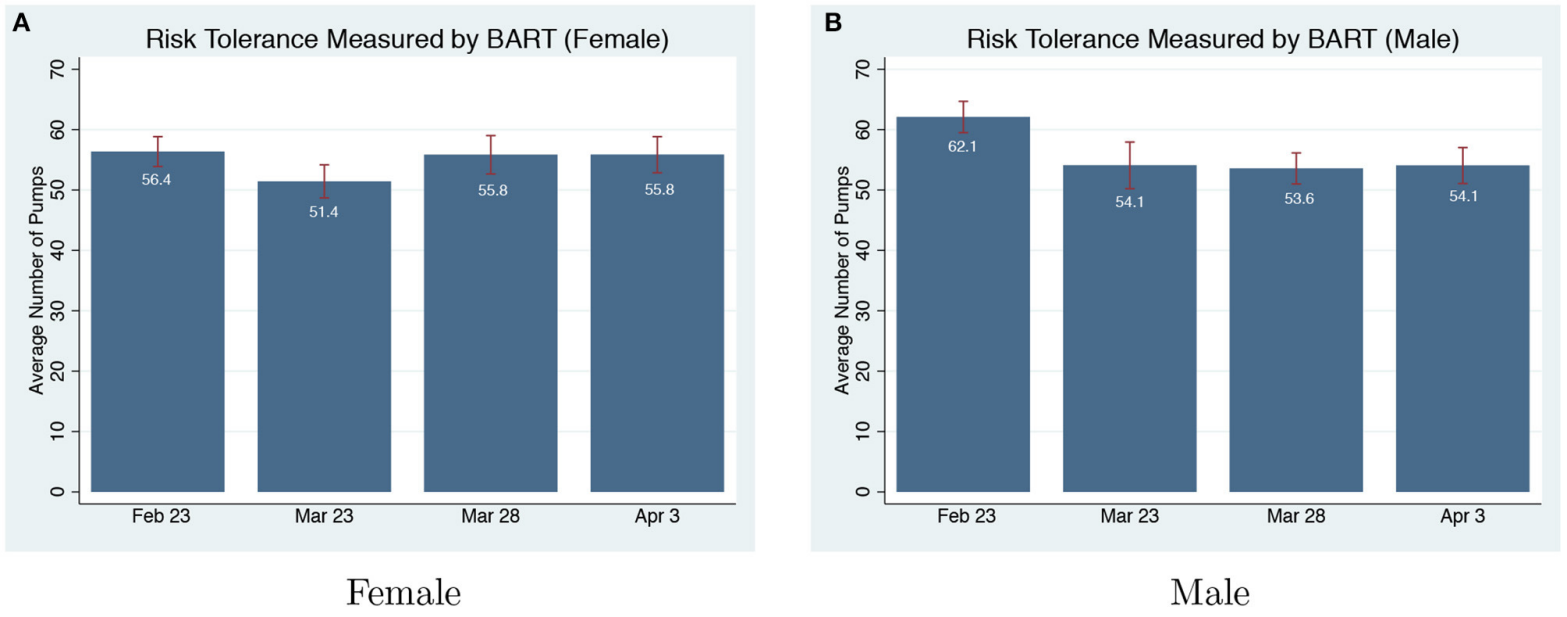
Risk-taking measured by Balloon Analogue Risk Task (BART) pre and on-pandemic. **(A)** Risk tolerance measured by BART (Female). **(B)** Risk tolerance measured by BART (Male).

We find that the change in risk-preference measured by BART is mainly contributed by changes in the risk-taking of man. Figure 1 shows that the risk-taking behavior of women does not change during the on-pandemic rounds compared to the pre-pandemic round (*p* = 0.236). However, men become significantly more risk-averse during the first on-pandemic round, and the changes are persistent for the remaining on-pandemic rounds. The difference in risk-taking behavior for men between the pre and on-pandemic rounds is significant (*p* = 0.001). We further find the change in men's risk-taking is overall swift, instead of just from a small subgroup. Table 3 compares the distribution of men in each pump range between pre-pandemic and on-pandemic rounds^6^. There is swift from pumps over 64 to pumps less than 64.

**Table 3:** Distribution of men in pump range.

	**Pumps [1, 32]**	**Pumps (32, 64]**	**Pumps (64, 96]**	**Pumps (96, 128]**
Pre-pandemic	0	53.7%	41.5%	4.9%
On-pandemic	14.2%	58.5%	25.5%	1.9%

**Figure 2:**
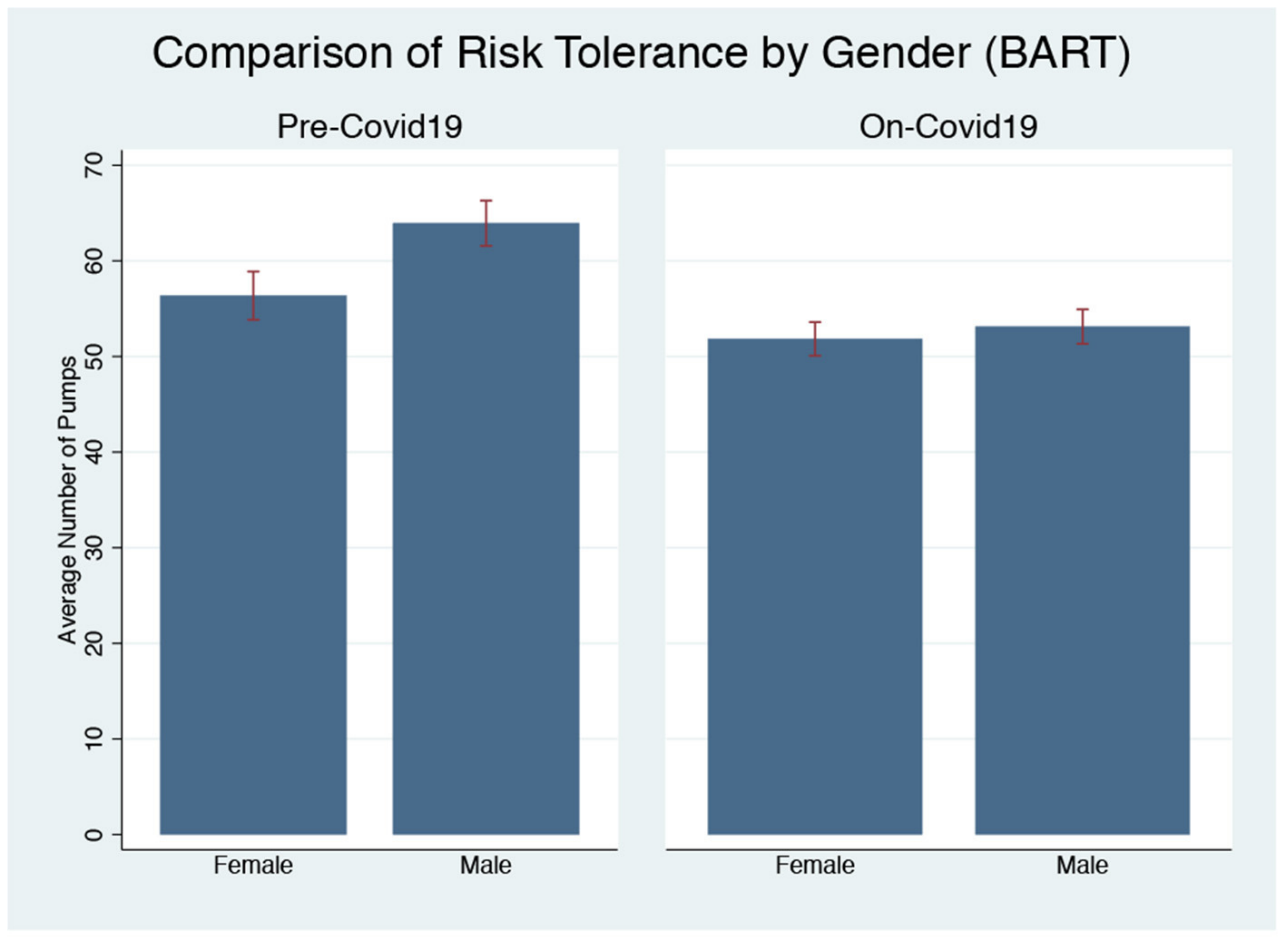
Gender differences in risk-taking by BART pre and on-pandemic.

Figure 2 shows that the gender gap in risk-taking behavior assessed by BART is present before COVID-19, with women being more risk averse than men (Mann-Whitney U-Test, *p* = 0.048). However, due to the reaction of men to COVID-19, the gender gap in risk aversion disappears during the on-pandemic rounds (*p* = 0.790). We further show that there are no significant differences by age (*p* = 0.612), education (*p* = 0.523), race (*p* = 0.443), or income (*p* = 0.342) between men and women. Thus, we argue that based on the BART results, men are more sensitive to COVID-19 in terms of the increased risk aversion compared to women.

**Figure 3:**
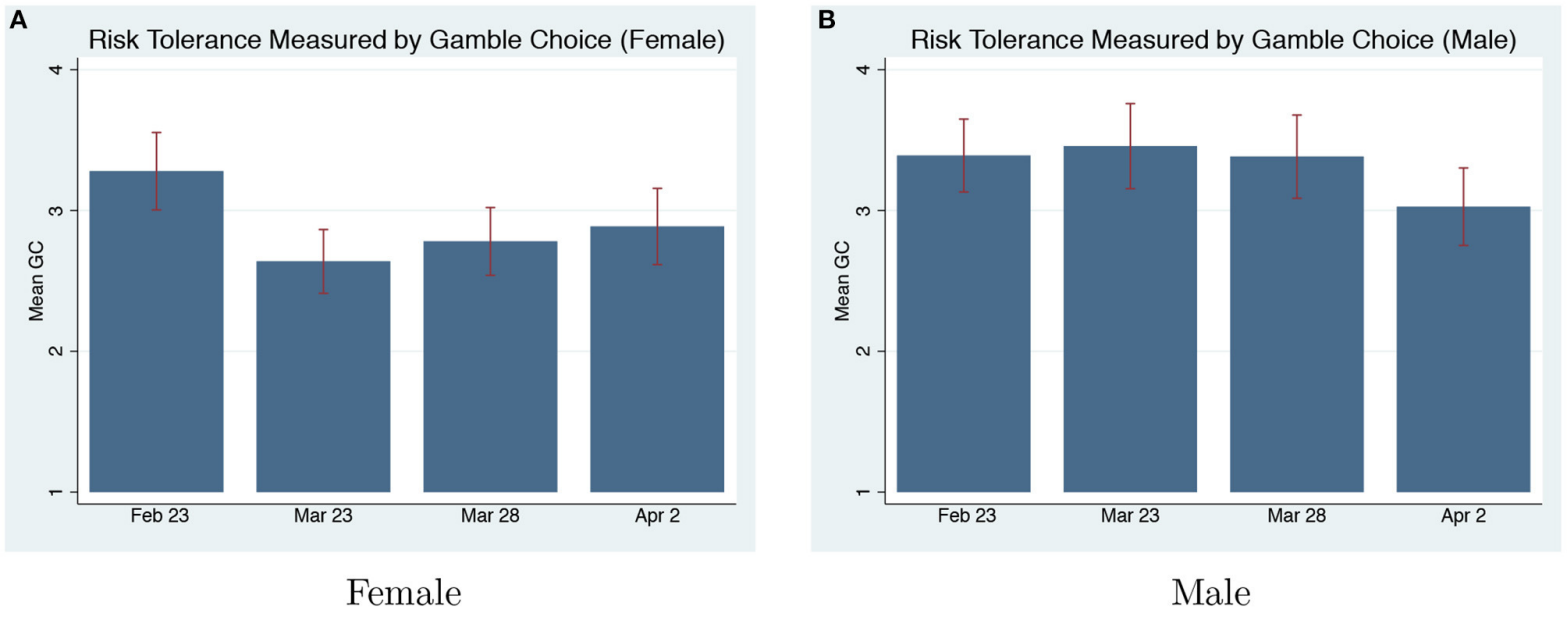
GC pre and on-pandemic. **(A)** Risk tolerance measured by gamble choice (Female). **(B)** Risk tolerance measured by gamble choice (Male).

**Gamble Choice**. We code the lottery 1 to 6 in the GC task as 1, 2, 3, 4, 5, 6 with 1 indicating extreme risk-aversion and 6 indicating risk-loving. We do not find any changes in risk attitudes elicited by the GC task for men (χ^2^ test, *p* = 0.854) or women (*p* = 0.381). Figure 3 shows the only change in risk-attitudes elicited by the GC is that women become more risk-averse during the first on-pandemic round, but the difference is only marginally significant (*p* = 0.097).

**Figure 4:**
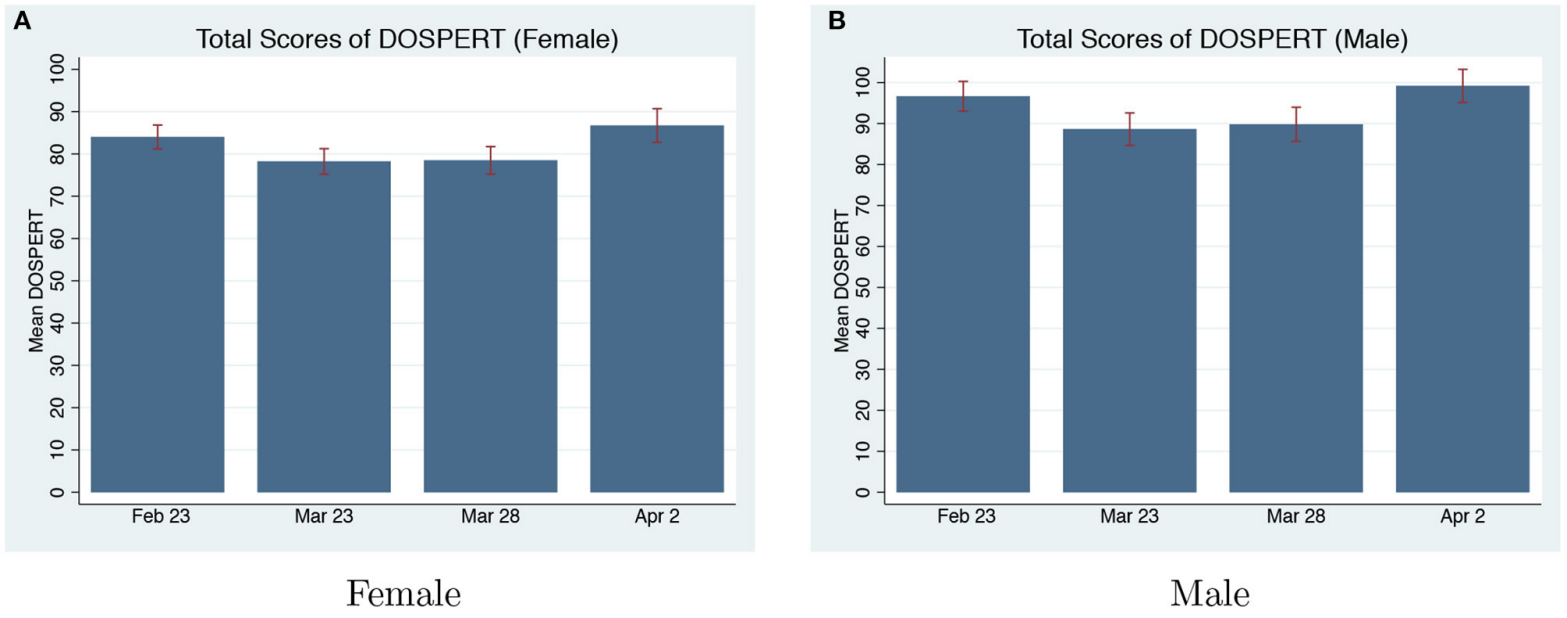
DOSPERT-Total Scores Pre and On-Pandemic. **(A)** Total scores of DOSPERT (Female). **(B)** Total scores of DOSPERT (Male).

**Domain-Specific Risk-Taking**. Figure 4 presents the DOSPERT-total scores of each round for both men and women. The only difference we find is between the score of the first on-pandemic round and the score of the pre-pandemic round for women, but the difference is marginal (Mann-Whitney U-Test, *p* = 0.076). In the following two on-pandemic rounds, women's DOSPERT-total scores returned to the pre-pandemic level (*p* = 0.176, *p* = 0.922). There are no differences in men's risk-taking between pre-pandemic and on-pandemic rounds based on DOSPERT-total scores (*p* = 0.105, *p* = 0.270, *p* = 0.814). When we combine the scores of all the three on-pandemic rounds and compare it with pre-pandemic, we do not find changes for both men and women (*p* = 0.309 for men; *p* = 0.209 for women).

We then test for differences for each sub-domain. Table 4 summarizes DOSPERT-total and sub-domain scores pre and on-pandemic separately by gender. The only statistical significant difference we find is women's scores on the Social domain. Women become more risk-averse during the pandemic in the Social domain (*p* = 0.003), with the changes starting in the first on-pandemic round (*p* = 0.042) and persistent for the remaining two on-pandemic rounds (*p* < 0.001, *p* = 0.099). In addition, the change is an overall swift of the distribution of females in each score range (refer to Appendix 9). The Social-domain in DOSPERT tests for the willingness to challenge social norms or social risky behavior such as confronting coworkers or family members. Social-domain in DOSPERT contains six items. We find the changes in womens' risk-preference mainly come from two items “Choosing a career that you truly enjoy over a more prestigious one” and “Starting a new career in your mid-thirties.” During COVID-19, many companies started to lay off workers, freeze hiring, and cut bonuses. The worries about losing jobs or being not able to find new jobs during the pandemic may cause people to become more risk-averse in the Social domain, especially for women based on our results. This is also in line with the literature about the gender layoff gap in the labor market during COVID-19 (Lin-Sperry, 2021).

**Table 4:** DOSPERT scores by gender.

	**Total**	**Ethical**	**Financial**	**Health/Safety**	**Recreational**	**Social**
Men (pre-pandemic)	96.66	13.73	18.37	18.56	16.39	29.61
Men (on-pandemic)	92.70	13.25	16.81	17.01	13.36	29.27
Women (pre-pandemic)	84.00	11.00	14.37	13.81	13.93	30.88
Women (on-pandemic)	81.12	11.58	14.04	13.95	14.03	27.52

**Figure 5:**
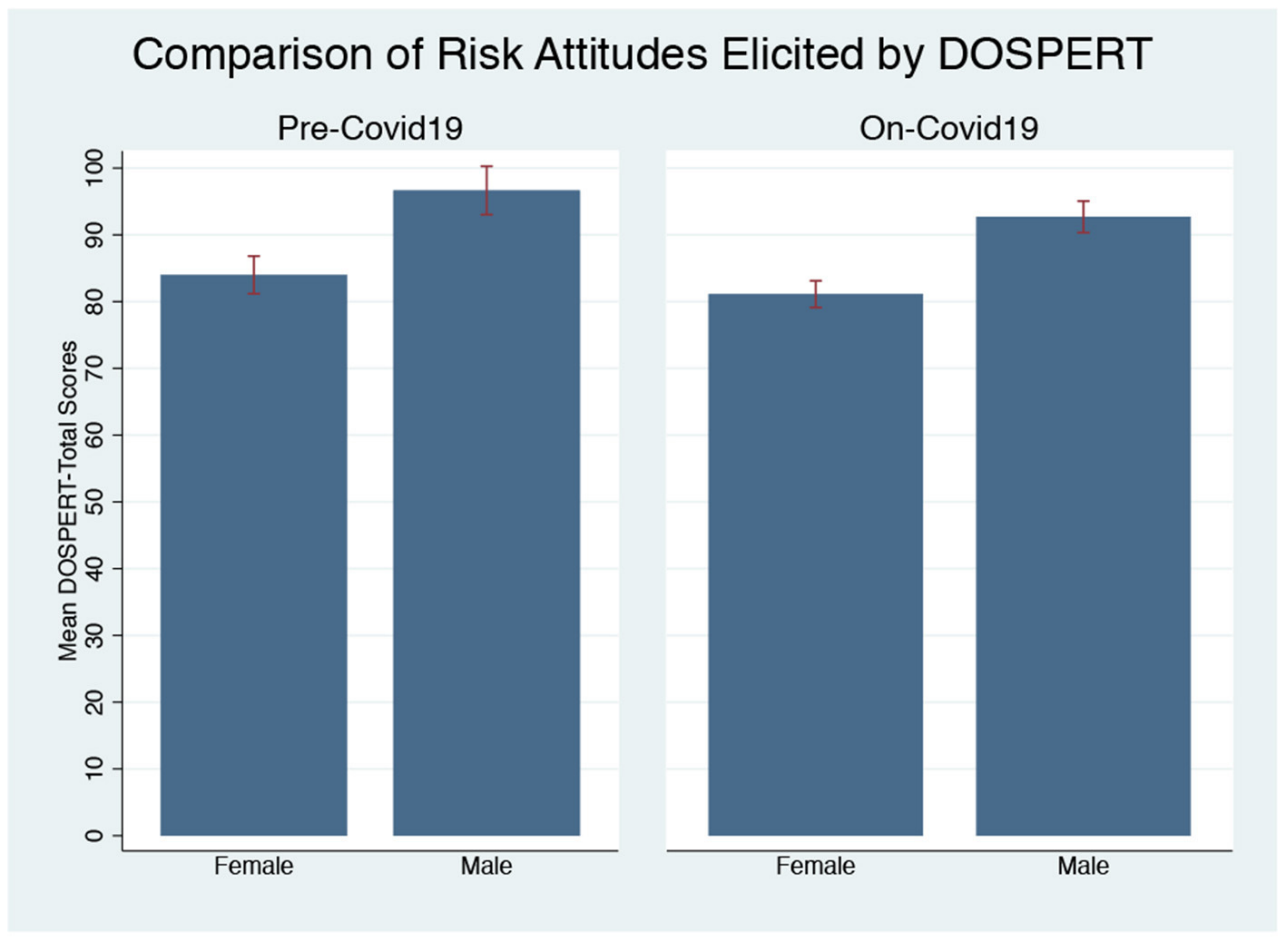
Gender differences in risk-taking by DOSPERT pre and on-pandemic.

Figure 5 shows the comparisons of DOSPERT-Total scores by gender. Based on the risk-attitudes elicited by DOSPERT, women are more risk-averse than men before COVID-19 (Mann-Whitney U-Test, *p* = 0.009). Since we only find changes in the Social sub-domain for women, and there is no significant change in the total score of men and women during COVID-19, there is still a gender gap in risk-attitudes elicited by DOSPERT during COVID-19 (*p* = 0.001).

**Sensation Seeking Scale**. The total score of SSS for women is significantly lower during the pandemic rounds compared to the pre-pandemic round (Mann-Whitney U-Test, *p* = 0.038). There is no significant change in men's SSS-Total or any sub-scale scores between the pre and on-pandemic rounds (refer to Table 5). The change in SSS-Total scores for women is mainly contributed by the sub-scale “Experience Seeking” (*p* < 0.001). “Experience Seeking” measures the desire to experience through travel and non-conforming lifestyles. COVID-19 makes women more risk-averse in the “Experience Seeking” aspect.

**Table 5:** SSS scores by gender.

	**Total**	**TAS**	**ES**	**DIS**	**BS**
Men (pre-pandemic)	16.32	3.83	5.07	4.51	2.90
Men (on-pandemic)	16.29	3.75	5.47	4.33	2.75
Women (pre-pandemic)	15.21	3.42	5.95	3.60	2.23
Women (on-pandemic)	12.88	3.00	4.42	3.17	2.29

**Figure 6:**
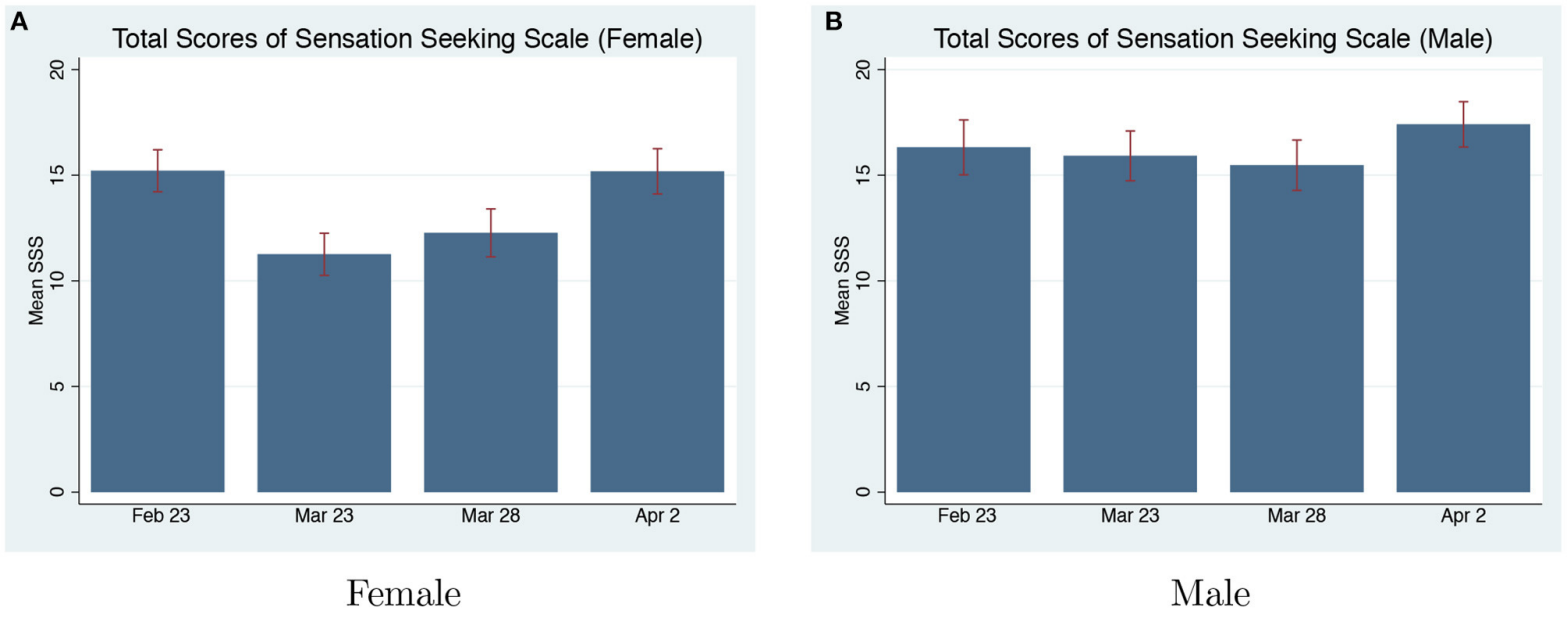
SSS-total scores pre and on-pandemic. **(A)** Total scores of sensation seeking scale (Female). **(B)** Total scores of sensation seeking scale (Male).

Figure 6 shows that the effects of COVID-19 on womens' risk-preference assessed by SSS are persistent for 2 weeks. There is a significant change when comparing women's risk-preference between the pre-pandemic and the first on-pandemic round (*p* = 0.005), and between the pre-pandemic and the second on-pandemic round (*p* = 0.033). On the third on-pandemic round (April 2), womens' risk-preference returned to the level before the pandemic (*p* = 0.939). Based on the self-reported SSS propensity measures, women become more risk-averse during COVID-19. However, after they get used to it, their risk-preference returns to the pre-pandemic level (refer to Appendix 10 for detailed changes in the distribution of women in each score range).

In Table 6, we estimate 6 regressions for the female and male separately (12 regressions in total). All regressions have the same dummy independent variable “On-pandemic.” We code the three on-pandemic rounds as 1, and the pre-pandemic round as 0. The dependent variable of each regression shown in each row of Table 6 is the measured outcomes by each method: the average number of pumps in BART, the choice in GC, and scores of DOSPERT and SSS. For BART, DOSPERT, and SSS, the regression models used are OLS; for GC, we use an ordered probit model. We control for age, education, income, and household size for all regressions^7^. The first four rows of Table 6 suggest that COVID-19 only has impacts on mens' risk-preference measured by BART. We further investigate the sub-domains of DOSPERT and SSS, and we only find women become more risk-averse in the “Social” domain of DOSPERT and “Experience Seeking” scale of SSS during COVID-19, which are shown in the last two rows of Table 6. All of these regression results are in line with the results presented above.

**Table 6:** Regression results by gender.

	**Female**	**Male**
	**On-Pandemic vs. Pre-Pandemic**	**On-Pandemic vs. Pre-Pandemic**
BART	–2.93 (3.45)	−11.04(3.34)^***^
GC	–0.29 (0.19)	–0.02 (0.20)
DOSPERT(total scores)	–1.92 (3.79)	–2.59 (4.45)
SSS(total scores)	–2.05 (1.24)	0.45 (1.33)
DOSPERT(social scores)	−3.23(1.22)^***^	–0.44 (1.21)
SSS(es scores)	−1.44(0.37)^***^	0.55 (0.43)
No. Observation	175	147

All regressions are controlled for age, education, income, and household size. Standard errors are in parentheses. ^*^p < 10%, ^**^p < 5%,

*** *p < 1%*.

To sum up, we find that based on GC, DOSPERT, and SSS total scores, both men's and women's risk-preference are stable during COVID-19. When we further investigate sub-domains/scales of DOSPERT and SSS, we find women become more risk-averse in the Social domain and the Experience Seeking scale. These results match our hypotheses about the stability of risk-preference based on different measures. The only result which conflicts our hypothesis is the increased risk aversion of men measured by BART. One possible explanation is that BART measures risk-taking by averaging choices over 30 balloons, instead of only based on one choice as in GC. The process of pumping repeated 30 balloons can be dynamic with the choice being affected by outcomes of previous balloons^8^. When different measuring processes interact with the pandemic, we may have different results regarding the stability of risk preferences. Thus, we suggest that more consideration needs to be given to the difference of measures within the same measurement tradition.

## 6. Correlation of Risk-Preference Measured by Different Methods

Risk preference evaluations have been shown to be context-dependent and low convergent validity across different elicitation techniques (refer to Reynaud and Couture, 2012; Crosetto and Filippin, 2016; Frey et al., 2017). Table 7 shows that in our experiment risk-preference measured by BART is positively correlated with risk-preference measured by the GC and SSS. The correlation of risk-preference measured by BART and DOSPERT total scores is not significant; however, the risk-preference measured by the Social sub-domain is significantly correlated with risk-preference measured in BART (Spearman's ρ = 0.107, *p* = 0.055). We find similar outcomes for risk-preference elicited by the GC, and it is significantly correlated with the risk-preference measured by the Financial sub-domain in DOSPERT (Spearman's ρ = 0.178, *p* = 0.001). The risk-preference measured by SSS is significantly correlated with all other three methods.

**Table 7:** Spearman's ρ of the correlations of risk-preference measured by four measures.

	**BART**	**Gamble choice**	**DOSPERT**	**SSS**
BART	1	0.211^***^	0.084	0.134^**^
Gamble Choice		1	0.089	0.144^***^
DOSPERT			1	0.688^***^
SSS				1

We use total scores for DOSPERT and SSS. ^*^p < 10%,

** *p < 5%*,

*** *p < 1%*.

We further test the degree of correlations in Table 7, and we have three arguments. First, all the correlations are positive, and this to some extent provides evidence of the validity of each method for assessing risk-preference. Second, the correlation between self-reported risk-preference (measured by DOSPERT and SSS) is stronger than the correlation between revealed risk-preference (elicited by BART and GC). This result is in line with the suggestions about lower convergent validity of revealed behavioral measures compared to self-reported propensity measures in both Frey et al. (2017) and Mata et al. (2018). Third, the correlations between two different types of measures are the weakest (BART/GC & DOSPERT/SSS), which is also suggested by Frey et al. (2017).

## 7. Conclusion

The COVID-19 pandemic is the worst crisis since World War II. It has affected and changed the life and work style of people all around the world. We believe people's risk-taking behavior is one of the most likely to be affected by the COVID-19 pandemic. We use four rounds of parallel experiments to test and track the dynamic changes in risk-preference during COVID-19. The first round was conducted around 1 month before COVID-19 started spreading in the United States, while the other three rounds were conducted after a state of emergency was declared.

We include four measures of risk-preference in each round. Two of them are revealed behavioral measures (BART and GC), and the other two are self-reported propensity measures (DOSPERT and SSS). The results are not consistent across all the four elicitation methods, and we also find heterogeneous effects for men and women. Men are more sensitive to the pandemic, and they become more risk-averse when measured by BART. The total score of DOSPERT and SSS show the stability of risk-preference for both men and women during COVID-19. We further test each sub-domain/scale of DOSPERT and SSS. We find that women become more risk-averse in the Social domain of DOSPERT and Experience Seeking scale of SSS. The GC does not show any changes in risk-preference for men or women. These results are in line with our hypotheses which are based on the discussion about the psychological construct of risk-preference. Our results show the caveat of testing risk-preference changes during COVID-19 using a single measure.

Our study provides new insights into the stability of risk-preference under pandemic shocks. In general, individuals' risk-preference is stable during COVID-19. However, when the measures capture risk-taking in some specific life domains and COVID-19 has stronger impacts on those domains (e.g., the social domain), the measures detect changes in risk-preference during COVID-19. This result differs by gender.

A limitation of our study is that we only track the changes through April 2, 2021. We find that womens' risk-attitudes elicited by SSS returned to the original level during the third on-pandemic round after becoming more risk-averse in the first two on-pandemic rounds. Future study may track the dynamic risk-preference changes for a longer period of time. The differential results suggested by BART and GC show that the same type of measures may also capture different factors of risk-preference. The heterogeneous effects between gender show women and men react differentially to the shock in terms of the changes of risk-taking behavior, or it suggests that the sensitivity of capturing risk-preference factors of measures might differ by gender. Future study about the construct of risk-preference should take differences between the same type of measures and gender into considerations.

## Data Availability Statement

The raw data supporting the conclusions of this article will be made available by the authors, without undue reservation.

## Ethics Statement

The studies involving human participants were reviewed and approved by IRB Administration Texas A&M University. The patients/participants provided their written informed consent to participate in this study.

## Author Contributions

PZ and MP: research idea and design and data analysis and interpretation, results, and manuscript drafting and writing. PZ: data collection and revisions. MP: language editing and appropriateness. Both authors contributed to the article and approved the submitted version.

## Conflict of Interest

The authors declare that the research was conducted in the absence of any commercial or financial relationships that could be construed as a potential conflict of interest.

## Publisher's Note

All claims expressed in this article are solely those of the authors and do not necessarily represent those of their affiliated organizations, or those of the publisher, the editors and the reviewers. Any product that may be evaluated in this article, or claim that may be made by its manufacturer, is not guaranteed or endorsed by the publisher.

## Footnotes

^1^The pre-pandemic data was part of another project (Zhang and Palma, 2021). Since we obtained a baseline at that time, this data served as the motivation for this study.

^2^The automatic version solves the data truncation problem of the original version in which the potential risk-taking behavior cannot be observed for the explosions. Pleskac et al. (2008) show that the automatic version does not change the validity of BART as an assessment of risk-taking.

^3^Our experiment was conducted on Amazon Mturk, and hence the payment of each task in our experiment is cent based.

^4^Mturk allows requesters to specify qualifications of workers for the HIT published. We required the location of workers to be in the United States, and the HIT approval rate and the number of HITs approved to be greater than 95% and 5,000, respectively.

^5^Although this is not the focus of this paper, there were no differences in the insurance purchasing rates across all four rounds.

^6^We separate the selected number of pumps into four ranges, as risk-neutral subjects would choose 64 pumps in BART by theory.

^7^The coefficients of “age” in the regressions for DOSPERT(total) and SSS(total) are negative and significant for both women and men. This means older people are more risk-averse based on DOSPERT and SSS measures. The coefficients of all the other control variables in all the regressions are not significant.

^8^In our automatic version, after entering the number of pumps, subjects need to wait to see the process of pumping (the balloon becoming larger and larger) and the outcome of the current balloon before they proceed to the next balloon.

## References

[B1] Abatayo A. L. Lynham J. (2020). Risk preferences after a typhoon: an artefactual field experiment with fishers in the Philippines. J. Econ. Psychol. 79:102195. 10.1016/j.joep.2019.102195

[B2] Anderson L. R. Mellor J. M. (2009). Are risk preferences stable? comparing an experimental measure with a validated survey-based measure. J. Risk Uncertainty 39, 137–160. 10.1007/s11166-009-9075-z

[B3] Angrisani M. Cipriani M. Guarino A. Kendall R. Ortiz de Zarate J. (2020). Risk Preferences at the Time of COVID-19: An Experiment With Professional Traders and Students. FRB of New York Staff Report 927.

[B4] Barrios J. M. Hochberg Y. (2020). Risk perception through the lens of politics in the time of the COVID-19 pandemic. Technical report, National Bureau of Economic Research.

[B5] Benjamin D. J. Cesarini D. Van Der Loos M. J. Dawes C. T. Koellinger P. D. Magnusson P. K.. (2012). The genetic architecture of economic and political preferences. Proc. Natl. Acad. Sci. U.S.A. 109, 8026–8031. 10.1073/pnas.1120666109 22566634 PMC3361436

[B6] Binder C. (2020). Coronavirus fears and macroeconomic expectations. Rev. Econ. Stat. 102, 721–730. 10.1162/rest_a_00931

[B7] Blais A.-R. Weber E. U. (2006). A domain-specific risk-taking (dospert) scale for adult populations. Judgm. Decis. Mak. 1, 33–47.

[B8] Bogliacino F. Codagnone C. Montealegre F. Folkvord F. Gómez C. Charris R.. (2021). Negative shocks predict change in cognitive function and preferences: assessing the negative affect and stress hypothesis. Sci. Rep. 11, 1–10. 10.1038/s41598-021-83089-0 33574445 PMC7878761

[B9] Callen M. Isaqzadeh M. Long J. D. Sprenger C. (2014). Violence and risk preference: experimental evidence from afghanistan. Am. Econ. Rev. 104, 123–148. 10.1257/aer.104.1.123

[B10] Chan H. F. Skali A. Savage D. A. Stadelmann D. Torgler B. (2020). Risk attitudes and human mobility during the COVID-19 pandemic. Sci. Rep. 10, 1–13. 10.1038/s41598-020-76763-2 33199737 PMC7669857

[B11] Charness G. Gneezy U. Imas A. (2013). Experimental methods: eliciting risk preferences. J. Econ. Behav. Organ. 87, 43–51. 10.1016/j.jebo.2012.12.023

[B12] Crosetto P. Filippin A. (2013). The bomb risk elicitation task. J. Risk Uncertain. 47, 31–65. 10.1007/s11166-013-9170-z

[B13] Crosetto P. Filippin A. (2016). A theoretical and experimental appraisal of four risk elicitation methods. Exp. Econ. 19, 613–641. 10.1007/s10683-015-9457-9

[B14] Dillon J. L. Scandizzo P. L. (1978). Risk attitudes of subsistence farmers in northeast brazil: a sampling approach. Am. J. Agric. Econ. 60, 425–435. 10.2307/1239939

[B15] Dohmen T. Falk A. Huffman D. Sunde U. Schupp J. Wagner G. G. (2011). Individual risk attitudes: measurement, determinants, and behavioral consequences. J. Eur. Econ. Assoc. 9, 522–550. 10.1111/j.1542-4774.2011.01015.x

[B16] Drichoutis A. C. Nayga R. M. (2021). On the stability of risk and time preferences amid the COVID-19 pandemic. Exp. Econ. 10.1007/s10683-021-09727-6. [Epub ahead of print]. 34404975 PMC8360830

[B17] Eckel C. C. El-Gamal M. A. Wilson R. K. (2009). Risk loving after the storm: a bayesian-network study of hurricane katrina evacuees. J. Econ. Behav. Organ. 69, 110–124. 10.1016/j.jebo.2007.08.012

[B18] Eckel C. C. Grossman P. J. (2008). Forecasting risk attitudes: an experimental study using actual and forecast gamble choices. J. Econ. Behav. Organ. 68, 1–17. 10.1016/j.jebo.2008.04.006

[B19] Eeckhoudt L. Gollier C. Schlesinger H. (1996). Changes in background risk and risk taking behavior. Econometrica 64, 683–689. 10.2307/2171866

[B20] Farnham A. Ziegler S. Blanke U. Stone E. Hatz C. Puhan M. A. (2018). Does the dospert scale predict risk-taking behaviour during travel? a study using smartphones. J. Travel Med. 25, tay064. 10.1093/jtm/tay064 30107438

[B21] Frey R. Pedroni A. Mata R. Rieskamp J. Hertwig R. (2017). Risk preference shares the psychometric structure of major psychological traits. Sci. Adv. 3, e1701381. 10.1126/sciadv.1701381 28983511 PMC5627985

[B22] Gassmann X. Malézieux A. Spiegelmanc E. Tisserand J.-C. (2020). Preferences after pandemics: Time and risk in the shadow of COVID-19. Technical report, Working Paper.

[B23] Gollier C. Pratt J. W. (1996). Risk vulnerability and the tempering effect of background risk. Econometrica 64, 1109–1123. 10.2307/2171958

[B24] Guenther B. Galizzi M. M. Sanders J. G. (2021). Heterogeneity in risk-taking during the COVID-19 pandemic: evidence from the uk lockdown. Front. Psychol. 12:852. 10.3389/fpsyg.2021.643653 33868115 PMC8046913

[B25] Guiso L. Paiella M. (2008). Risk aversion, wealth, and background risk. J. Eur. Econ. Assoc. 6, 1109–1150. 10.1162/JEEA.2008.6.6.110912071209

[B26] Hanaoka C. Shigeoka H. Watanabe Y. (2018). Do risk preferences change? evidence from the great east japan earthquake. Am. Econ. J. 10, 298–330. 10.1257/app.20170048

[B27] Harrison G. W. Lau M. I. Yoo H. I. (2020). Risk attitudes, sample selection, and attrition in a longitudinal field experiment. Rev. Econ. Stat. 102, 552–568. 10.1162/rest_a_00845

[B28] Highhouse S. Nye C. D. Zhang D. C. Rada T. B. (2017). Structure of the dospert: Is there evidence for a general risk factor? J. Behav. Decis Mak. 30, 400–406. 10.1002/bdm.195325855820

[B29] Holt C. A. Laury S. K. (2002). Risk aversion and incentive effects. Am. Econ. Rev. 92, 1644–1655. 10.1257/000282802762024700

[B30] Inquisit (2016). Computer Software. Available online at: http://www.millisecond.com.

[B31] Jakiela P. Ozier O. (2019). The impact of violence on individual risk preferences: evidence from a natural experiment. Rev. Econ. Stat. 101, 547–559. 10.1162/rest_a_00763

[B32] Jetter M. Magnusson L. M. Roth S. (2020). Becoming sensitive: Males? risk and time preferences after the 2008 financial crisis. Eur. Econ. Rev. 128:103512. 10.1016/j.euroecorev.2020.103512

[B33] Kahsay G. A. Osberghaus D. (2018). Storm damage and risk preferences: panel evidence from germany. Environ. Resour. Econ. 71, 301–318. 10.1007/s10640-017-0152-5

[B34] Kawohl W. Nordt C. (2020). COVID-19, unemployment, and suicide. Lancet Psychiatry 7, 389–390. 10.1016/S2215-0366(20)30141-3 32353269 PMC7185950

[B35] Lee J. (2008). The effect of the background risk in a simple chance improving decision model. J. Risk Uncertain 36, 19–41. 10.1007/s11166-007-9028-3

[B36] Lejuez C. W. Read J. P. Kahler C. W. Richards J. B. Ramsey S. E. Stuart G. L.. (2002). Evaluation of a behavioral measure of risk taking: the balloon analogue risk task (bart). J. Exp. Psychol. 8, 75. 10.1037/1076-898X.8.2.75 12075692

[B37] Li Z. Lin P.-H. Kong S.-Y. Wang D. Duffy J. (2021). Conducting large, repeated, multi-game economic experiments using mobile platforms. PLoS ONE 16:e0250668. 10.1371/journal.pone.0250668 33914785 PMC8084158

[B38] Lin-Sperry E. (2021). Covid19 Recession: Gender Layoff Gap Explodes. Berkeley, CA: University of California.

[B39] Lohmann P. Gsottbauer E. You J. Kontoleon A. (2020). Social Preferences and Economic Decision-Making in the Wake of COVID-19: Experimental Evidence From China. Available at SSRN 3705264. 10.1016/j.jebo.2022.12.007 PMC9744689 36531911

[B40] Marianne B. (2011). New perspectives on gender. Handbook Labor Econ. 4, 1543–1590. 10.1016/S0169-7218(11)02415-4

[B41] Mata R. Frey R. Richter D. Schupp J. Hertwig R. (2018). Risk preference: a view from psychology. J. Econ. Perspect. 32, 155–172. 10.1257/jep.32.2.155 30203934

[B42] Niederle M. Vesterlund L. (2007). Do women shy away from competition? do men compete too much? Q. J. Econ. 122, 1067–1101. 10.1162/qjec.122.3.106733869490

[B43] Officer R. R. Halter A. N. (1968). Utility analysis in a practical setting. Am. J. Agric. Econ. 50, 257–277. 10.2307/1237541

[B44] Page L. Savage D. A. Torgler B. (2014). Variation in risk seeking behaviour following large losses: a natural experiment. Eur. Econ. Rev. 71, 121–131. 10.1016/j.euroecorev.2014.04.009

[B45] Pleskac T. J. Wallsten T. S. Wang P. Lejuez C. (2008). Development of an automatic response mode to improve the clinical utility of sequential risk-taking tasks. Exp. Clin. Psychopharmacol. 16, 555. 10.1037/a0014245 19086776

[B46] Quiggin J. (2003). Background risk in generalized expected utility theory. Econ. Theory 22, 607–611. 10.1007/s00199-002-0311-x

[B47] Reynaud A. Couture S. (2012). Stability of risk preference measures: results from a field experiment on french farmers. Theory Decis. 73, 203–221. 10.1007/s11238-012-9296-5

[B48] Schildberg-Hörisch H. (2018). Are risk preferences stable? J. Econ. Perspect. 32, 135–154. 10.1257/jep.32.2.135 30203933

[B49] Shachat J. Walker M. J. Wei L. (2021a). How the onset of the COVID-19 pandemic impacted pro-social behaviour and individual preferences: Experimental evidence from china. J. Econ. Behav. Organ. 190, 480–494. 10.1016/j.jebo.2021.08.001 34642514 PMC8494513

[B50] Shachat J. Walker M. J. Wei L. (2021b). The impact of an epidemic: Experimental evidence on preference stability from wuhan. AEA Pap. Proc. 111, 302–306. 10.1257/pandp.20211002

[B51] Shou Y. Olney J. (2020). Assessing a domain-specific risk-taking construct: a meta-analysis of reliability of the DOSPERT scale. Judg. Decis. Mak. 15, 112–134.

[B52] Shurchkov O. Eckel C. C. (2018). Gender Differences in Behavioral Traits and Labor Market Outcomes. Oxford, UK: Oxford University Press.

[B53] Stigler G. J. Becker G. S. (1977). De gustibus non est disputandum. Am. Econ. Rev. 67, 76–90.11300341

[B54] Tsetlin I. Winkler R. L. (2005). Risky choices and correlated background risk. Manag. Sci. 51, 1336–1345. 10.1287/mnsc.1050.0414

[B55] Wong A. Carducci B. J. (1991). Sensation seeking and financial risk taking in everyday money matters. J. Bus. Psychol. 5, 525–530. 10.1007/BF01014500

[B56] Zaleski Z. (1984). Sensation-seeking and risk-taking behaviour. Pers. Individ. Dif. 5, 607–608. 10.1016/0191-8869(84)90039-4

[B57] Zhang P. Palma M. A. (2021). Compulsory versus voluntary insurance: an online experiment. Am. J. Agric. Econ. 103, 106–125. 10.1111/ajae.12120

[B58] Zhong S. Chew S. H. Set E. Zhang J. Xue H. Sham P. C.. (2009). The heritability of attitude toward economic risk. Twin Res. Hum. Genet. 12, 103–107. 10.1375/twin.12.1.103 19210185

[B59] Zoumpourlis V. Goulielmaki M. Rizos E. Baliou S. Spandidos D. A. (2020). [comment] the COVID-19 pandemic as a scientific and social challenge in the 21st century. Mol. Med. Rep. 22, 3035–3048. 10.3892/mmr.2020.11393 32945405 PMC7453598

[B60] Zuckerman M. (1994). Behavioral Expressions and Biosocial Bases of Sensation Seeking. Cambridge: Cambridge University Press.

[B61] Zuckerman M. (2007). Sensation seeking and risky behavior. Am. Psychol. Assoc. 10.1037/11555-000

[B62] Zuckerman M. Eysenck S. B. Eysenck H. J. (1978). Sensation seeking in england and america: cross-cultural, age, and sex comparisons. J. Consult. Clin. Psychol. 46, 139. 10.1037/0022-006X.46.1.139 627648

[B63] Zuckerman M. Kolin E. A. Price L. Zoob I. (1964). Development of a sensation-seeking scale. J. Consult. Psychol. 28, 477. 10.1037/h0040995 14242306

