# Supplementary material for: Stability of Risk Preferences During COVID-19: Evidence From Four Measurements
Source: Front Psychol. 2022 Feb 10;12:702028. doi: 10.3389/fpsyg.2021.702028 (PMC8868569; doi:10.3389/fpsyg.2021.702028)
Supplement: Supplementary file 1 [file Presentation_1.pdf]

Appendix for Stability of Risk Preferences During COVID-19: Evidence from Four Measurements

Peilu Zhang Marco A. Palma

December, 2021

The material contained herein is supplementary to the article named in the title and published in the Frontiers in Psychology.

A1. Daily Trends in the Number of COVID-19 Cases in The United States Reported to CDC (`https://covid.cdc.gov/covid-data-tracker`)

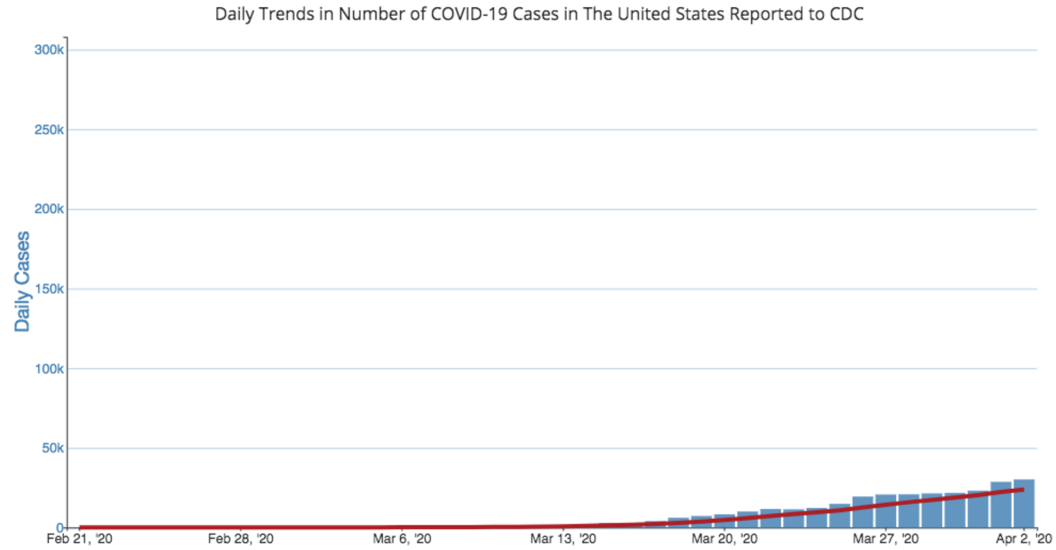

Figure A1

A2. Algorithm of explosion probability of BART.

In BART, the arrangement of the probability of explosion of each balloon is determined by the balloon's maximum possible number of pump. In our experiment, the maximum possible number of pump of each balloon is 128.[1] This means the probability of explosion at the first pump is 1/128. If the balloon did not explode after the first pump, the probability of explosion at the second pump is 1/127; 1/126 at the third pump, and so on up until the 128th pump, at which the balloon will explode for sure. Based on this explosion algorithm, the expected explosion point is the 64th pump. The expected earnings for each balloon are a bow-shaped function with a maximum at the 64th pump.

The decisions of participants on each balloon can be formalized as the decisions on the lotteries:

$$L = \begin{cases} 0 & \textit{probability} : k/128 = 1 - (128-k)/128 \\ \gamma k & \textit{probability} : (128-k)/128 = (127/128 * 126/127 * \ldots * (128-k)/(129-k)) \end{cases}$$

where k is the number of possible pumps which in our experiment $\in [0, 128]$, while $\gamma > 0$ is a scale factor. The expected value of these lotteries is equal to $\gamma(128k - k^2)/128$. Then, the BART allows for the estimation of the coefficient of risk aversion by assuming a constant relative risk aversion (CRRA) utility function $u(k) = k^r$. A *risk-averse* would choose less than 64 pumps; a *risk-neutral* individual would choose $k^* = 64$; a *risk-seeking* participant would choose more than 64 pumps.

[1]In Lejuez et al. (2002), the significant correlation of the BART with self-reported risk behavior in the real world occurred only with data from the balloon with a maximum number of 128 pumps.

A3. DOSPERT.

For each of the following statements, please indicate the likelihood that you would engage in the described activity or behavior if you were to find yourself in that situation. Provide a rating from Extremely Unlikely to Extremely Likely.

(1). Admitting that your tastes are different from those of a friend. (S)

(2). Going camping in the wilderness. (R)

(3). Betting a day's income at the horse races. (F)

(4). Investing 10% of your annual income in a moderate growth mutual fund. (F)

(5). Drinking heavily at a social function. (H/S)

(6). Taking some questionable deductions on your income tax return. (E)

(7). Disagreeing with an authority figure on a major issue. (S)

(8). Betting a day's income at a high-stake poker game. (F)

(9). Having an affair with a married man/woman. (E)

(10). Passing off somebody else's work as your own. (E)

(11). Going down a ski run that is beyond your ability. (R)

(12). Investing 5% of your annual income in a very speculative stock. (F)

(13). Going whitewater rafting at high water in the spring. (R)

(14). Betting a day's income on the outcome of a sporting event. (F)

(15). Engaging in unprotected sex. (H/S)

(16). Revealing a friend's secret to someone else. (E)

(17). Driving a car without wearing a seat belt. (H/S)

(18). Investing 10% of your annual income in a new business venture. (F)

(19). Taking a skydiving class. (R)

(20). Riding a motorcycle without a helmet. (H/S)

(21). Choosing a career that you truly enjoy over a more prestigious one. (S)

(22). Speaking your mind about an unpopular issue in a meeting at work. (S)

(23). Sunbathing without sunscreen. (H/S)

(24). Bungee jumping off a tall bridge. (R)

(25). Piloting a small plane. (R)

(26). Walking home alone at night in an unsafe area of town. (H/S)

(27). Moving to a city far away from your extended family. (S)

(28). Starting a new career in your mid-thirties. (S)

(29). Leaving your young children alone at home while running an errand. (E)

(30). Not returning a wallet you found that contains $200. (E)

Note: E = Ethical, F = Financial, H/S = Health/Safety, R = Recreational, and S = Social.

A4. SSS.

Each of the items below contains two choices, A and B. Please click the letter of the choice which most describes your likes or the way you feel. In some cases you may find items in which both choices describe your likes or feelings. Please choose the one which better describes your likes or feelings. In some cases you may find items in which you do not like either choice. In these cases mark the choice you dislike least. Do not leave any items blank.

In this part, there are not right or wrong answers. Be frank and give your honest appraisal of yourself.

(1) A. I like "wild" uninhibited parties

B. I prefer quiet parties with good conversation

(2) A. There are some movies I enjoy seeing a second or even a third time

B. I can't stand watching a movie that I've seen before

(3) A. I often wish I could be a mountain climber

B. I can't understand people who risk their necks climbing mountains

(4) A. I dislike all body odors

B. I like some for the earthly body smells

(5) A. I get bored seeing the same old faces

B. I like to comfortable familiarity of everyday friends

(6) A. I like to explore a strange city or section of town by myself, even if it means getting lost

B. I prefer a guide when I am in a place I don't know well

(7) A. I dislike people who do or say things just to shock or upset others

B. When you can predict almost everything a person will do and say he or she must be a bore

(8) A. I usually don't enjoy a movie or play where I can predict what will happen in advance

B. I don't mind watching a movie or a play where I can predict what will happen in advance

(9) A. I have tried marijuana or would like to

B. I would never smoke marijuana

(10) A. I would not like to try any drug which might produce strange and dangerous effects on me

B. I would like to try some of the new drugs that produce hallucinations

(11) A. A sensible person avoids activities that are dangerous

B. I sometimes like to do things that are a little frightening

(12) A. I dislike "swingers" (people who are uninhibited and free about sex)

B. I enjoy the company of real "swingers"

(13) A. I find that stimulants make me uncomfortable

B. I often like to get high (drinking liquor or smoking marijuana)

(14) A. I like to try new foods that I have never tasted before

B. I order the dishes with which I am familiar, so as to avoid disappointment and unpleasantness

(15) A. I enjoy looking at home movies or travel slides

B. Looking at someone's home movies or travel slides bores me tremendously

(16) A. I would like to take up the sport of water skiing

B. I would not like to take up water skiing

(17) A. I would like to try surf boarding

B. I would not like to try surf boarding

(18) A. I would like to take off on a trip with no preplanned or definite routes, or timetable

B. When I go on a trip I like to plan my route and timetable fairly carefully

(19) A. I prefer the "down to earth" kinds of people as friends

B. I would like to make friends in some of the "far out" groups like artists or "punks"

(20) A. I would not like to learn to fly an airplane

B. I would like to learn to fly an airplane

(21) A. I prefer the surface of the water to the depths

B. I would like to go scuba diving

(22) A. I would like to meet some persons who are homosexual (men or women)

B. I stay away from anyone I suspect of being "gay or lesbian"

(23) A. I would like to try parachute jumping

B. I would never want to try jumping out of a plane with or without a parachute

(24) A. I prefer friends who are excitingly unpredictable

B. I prefer friends who are reliable and predictable

(25) A. I am not interested in experience for its own sake

B. I like to have new and exciting experiences and sensations even if they are a little frightening, unconventional, or illegal

(26) A. The essence of good art is in its clarity, symmetry of form and harmony of colors

B. I often find beauty in the "clashing" colors and irregular forms of modern paintings

(27) A. I enjoy spending time in the familiar surroundings of home

B. I get very restless if I have to stay around home for any length of time

(28) A. I like to dive off the high board

B. I don't like the feeling I get standing on the high board (or I don't go near it at all)

(29) A. I like to date members of the opposite sex who are physically exciting

B. I like to date members of the opposite sex who share my values

(30) A. Heavy drinking usually ruins a party because some people get loud and boisterous

B. Keeping the drinks full is the key to a good party

(31) A. The worst social sin is to be rude

B. The worst social sin is to be a bore

(32) A. A person should have considerable sexual experience before marriage

B. It's better if two married persons begin their sexual experience with each other

(33) A. Even if I had the money I would not care to associate with flight rich persons like those in the "jet set"

B. I could conceive of myself seeking pleasures around the world with the "jet set"

(34) A. I like people who are sharp and witty even if they do sometimes insult others

B. I dislike people who have their fun at the expense of hurting the feelings of others

(35) A. There is altogether too much portrayal of sex in movies

B. I enjoy watching many of the "sexy" scenes in movies

(36) A. I feel best after taking a couple of drinks

B. Something is wrong with people who need liquor to feel good

(37) A. People should dress according to some standard of taste, neatness, and style

B. People should dress in individual ways even if the effects are sometimes strange

(38) A. Sailing long distances in small sailing crafts is foolhardy

B. I would like to sail a long distance in a small but seaworthy sailing craft

(39) A. I have no patience with dull or boring persons

B. I find something interesting in almost every person I talk to

(40) A. Skiing down a high mountain slope is a good way to end up on crutches

B. I think I would enjoy the sensations of skiing very fast down a high mountain slope

A5. Gamble Choice.

In this part, please make a choice among the following six gamble choices. Each choice has two events A and B, and each event's chance of occurring is 50%. After you make your choice, the system will randomly choose an event and depending on your choice, you will get the corresponding payoff for that event.
If you select a gamble with a negative payoff for outcome B, negative payoffs will be deducted from your payment in previous parts of the experiment.

Gamble choice	The event	Probability	Payoff (cents)
1.	A	50%	10
	B	50%	10
2.	A	50%	18
	B	50%	6
3.	A	50%	26
	B	50%	2
4.	A	50%	34
	B	50%	-2
5.	A	50%	42
	B	50%	-6
6.	A	50%	44
	B	50%	-8

Continue

Figure A2: Screenshot of the gamble-choice task.

A6. Demographic Characteristics.

Table A1: Demographic characteristics

	Pre-pandemic	On-pandemic	P-values
Age (mean)	39	42	0.061
Gender	51.2% (F)<br>48.8% (M)	55.5% (F)<br>44.5% (M)	0.499
Race (White)	78.6%	79.4%	0.321
Household(mean)	2.5	2.8	0.081
Education(median)	4 year/Bachelor's Degree	4 year/Bachelor's Degree	0.114
Income(mean)	\$50,000 - \$59,999	\$50,000 - \$59,999	0.734

Notes: The p-values in the last column from two-sided Mann-Whitney U-Tests (Age, Household, Income) and χ^2 tests (Gender, Race, Education).

A7. Distribution of women in each DOSPERT-Social and SSS-ES score range.

Table A2: Distribution of Women in DOSPERT-Social Score Range

	Scores [1, 14]	Scores [15, 28]	Scores [29, 42]
Pre-pandemic	2.3%	30.2%	67.4%
On-pandemic	3.0%	56.1%	40.9%

Table A3: Distribution of Women in SSS-ES Score Range

	Scores [0, 2]	Scores [3, 6]	Scores [7, 10]
Pre-pandemic	0	62.8%	37.2%
On-pandemic	22.7%	59.1%	18.2%

A8. Instructions.

(1). General Instructions

Now you will be presented with 30 balloons in the computer screen.

You have to decide how many times you want to pump each balloon. For every successful pump you will earn money. However, the explosion point for each balloon is random. The maximum possible number of pumps for each balloon is 128. The explosion point is random and it can be anywhere in the range from the first (1st) to the last (128th) pump.

For each balloon, you will be asked to select how many times you want to pump it up. You get a MONETARY reward of $0.01 for every successful pump. HOWEVER, if a balloon explodes before it reaches the number of pumps you indicated, you earn $0.00 for that balloon.

After each trial, a new balloon will appear.

For SOME balloons, you have an opportunity to buy an Insurance to protect yourself against the risk of an explosion for that particular balloon. Please make your decisions carefully.

At the end of the experiment, 3 balloons will be RANDOMLY SELECTED, and you will be paid the amount of money earned for these three balloons.

(2). Summary

* You write the number of times you want to pump up each balloon in a provided textbox.

* Remember: each balloon can be pumped up to 128 times (it will surely pop at 128th pump).

* Each balloon is then pumped up until a) that number is reached or b) it pops. Whatever occurs first.

* If it does not explode, you make $0.01 for each pump.

* If it does explode, you will not make any money on that balloon.

* There are a total of 30 balloons.

* Only some balloons have the opportunity to purchase insurance. (if Purely Voluntary

or Mixed Treatment)

For some balloons, you are REQUIRED to buy an Insurance. (if Purely Compulsory Treatment)

* At the end, you will be paid the exact amount you earned on THREE randomly selected balloons.

Continue when you are ready to start.

(3).Insurance

On the following balloon, you have an opportunity to buy an insurance to protect yourself against the risk of explosion. The price of the insurance is $0.40.

If the balloon does explode, the insurance will pay you $0.64; if the balloon does not explode, the insurance will pay you nothing, and the cost is not refunded. However, you will keep the earnings you make in that balloon if it is selected at the end of the experiment.

Remember: each balloon can be pumped up to 128 times;

The insurance is only valid for this balloon.

Before proceeding to make your choices, you have to correctly answer the following three questions.

(1). If you choose to buy the insurance, and you pump 128 times, then how much would you earn for this balloon?

A. $0 B. $64 C. $24 D. $40

(2). If you choose NOT to buy the insurance, and you pump 64 times, and the balloon does not explode, then how much would you earn for this balloon?

A. $64 B. $24 C. $0 D. $40

(3). If you choose to buy the insurance, and you pump 70 times, and the balloon does not explode, then how much would you earn for this balloon?

A. $70 B. $30 C. $0 D. $40

(4a). Insurance choice

Now please indicate your decision by Clicking the options below.

Yes, I buy the insurance at a cost of $0.40.

No, I do not buy the insurance.

(5). BART

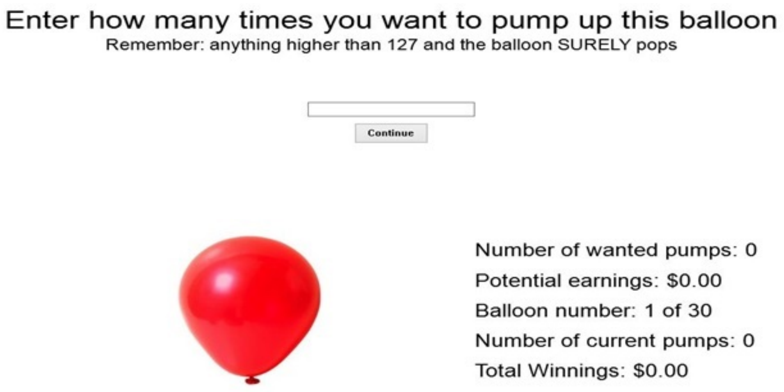

Figure A3: Screenshot of BART.

(6). Earnings in BART

Congratulations!

Your earnings on 1-30 balloons are(),(),()...respectively, by randomly selecting three of them, your payment of this part is $().

The balloon task is now complete. Please Click continue to go to the next part.

A9. Demographic survey.

Please answer the following survey questions.

(1) Please enter your age in years.

(2) Please indicate the HIGHEST level of education you have completed.

Some High School or less

High School Diploma

Some College

2 year/Associates Degree

4 year/Bachelor's Degree

Some Graduate School

Graduate Degree

(3) Including yourself, how many people live in your household?

(4) Please indicate your gender.

Male

Female

(5) Please indicate your race.

Asian/ Pacific Islander

African American

Caucasian/ White

Native American/ Indigenous

Hispanic

Other (Please list below)

(6) Please indicate your household yearly income for 2020. (Include all forms of income, including salary, interest and dividend payments, tips, scholarship support, student loans, parental support, and allowance)

Less than $30,000

$30,000 - $39,999

$40,000 - $49,999

$50,000 - $59,999

$60,000 - $69,999

$70,000 - $79,999

$80,000 - $89,999

$90,000 - $99,999

$100,000 - $149,999

$150,000 or more

(7) Are you (/have you been) in self-isolation due to coronavirus recently?

Yes.

No.

(8) Do you think the Insurance Option in the balloon task is clear?

Yes.

No.
